# Expanding the environmental scope: an environment-wide association study for mental well-being

**DOI:** 10.1038/s41370-021-00346-0

**Published:** 2021-06-14

**Authors:** Margot P. van de Weijer, Bart M. L. Baselmans, Jouke-Jan Hottenga, Conor V. Dolan, Gonneke Willemsen, Meike Bartels

**Affiliations:** 1 Department of Biological Psychology, Faculty of Behavioural and Movement Sciences, Vrije Universiteit Amsterdam, Amsterdam, The Netherlands; 2 Amsterdam Public Health Research Institute, Amsterdam University Medical Centre, Amsterdam, The Netherlands; 3 Institute for Molecular Bioscience, The University of Queensland, Brisbane, QLD Australia

**Keywords:** Well-being, Environment, Environment-wide association, Polygenic score, Safety, Socioeconomic status

## Abstract

**Background:**

Identifying modifiable factors associated with well-being is of increased interest for public policy guidance. Developments in record linkage make it possible to identify what contributes to well-being from a myriad of factors. To this end, we link two large-scale data resources; the Geoscience and Health Cohort Consortium, a collection of geo-data, and the Netherlands Twin Register, which holds population-based well-being data.

**Objective:**

We perform an Environment-Wide Association Study (EnWAS), where we examine 139 neighbourhood-level environmental exposures in relation to well-being.

**Methods:**

First, we performed a generalized estimation equation regression (*N* = 11,975) to test for the effects of environmental exposures on well-being. Second, to account for multicollinearity amongst exposures, we performed principal component regression. Finally, using a genetically informative design, we examined whether environmental exposure is driven by genetic predisposition for well-being.

**Results:**

We identified 21 environmental factors that were associated with well-being in the domains: housing stock, income, core neighbourhood characteristics, livability, and socioeconomic status. Of these associations, socioeconomic status and safety are indicated as the most important factors to explain differences in well-being. No evidence of gene-environment correlation was found.

**Significance:**

These observed associations, especially neighbourhood safety, could be informative for policy makers and provide public policy guidance to improve well-being. Our results show that linking databases is a fruitful exercise to identify determinants of mental health that would remain unknown by a more unilateral approach.

## Introduction

Demographic factors are widely recognized as important for people’s functioning and mental health. For example, urbanization, i.e., the movement of population from rural to more urbanized areas, is accompanied by both beneficial and detrimental effects on mental health. Urbanization is often associated with economic growth and prosperity [1, 2], and comes with better infrastructure and better access to health care services [3]. Mental disorders, though, are more prevalent in more urbanized areas [4, 5] for example due to less access to green space [6], increased social stress [7], and less (perceived) neighbourhood safety [8, 9]. Moreover, genetic factors influence where people prefer to live and how their environment impacts them. For instance, research into urbanization and schizophrenia showed that individuals with a higher genetic predisposition to schizophrenia tend to live in urbanized areas. For instance, research into urbanization and schizophrenia showed that individuals with increased genetic predisposition for schizophrenia tend to live in more urbanized areas. While it was previously assumed that the higher schizophrenia prevalence was explained by increased environmental stress in urbanized areas, this study revealed that part of why schizophrenia is more prevalent in cities is because of an increased genetic predisposition [10].

Recent developments in data sharing and linkage are transforming the way we approach mental health topics and its possible correlates. One of the developments that makes it possible to identify what contributes to mental health and human functioning from a myriad of factors is record linkage. By linking large data resources that contain different types of information, novel, otherwise invisible patterns can be uncovered. A well-known example in is the UK Biobank. By linking genetic (and biological, phenotypic) data to existing health records, great advances have been made in identifying risk factors for disorders such as schizophrenia and depression [11–13]. Record linkage is becoming increasingly accessible for researchers across different disciplines and countries. For example, in the Netherlands, data on households, job benefits, education, crime, and more is available on a population-based scale [14]. This type of data can, under certain conditions and strict privacy regulations, be linked to patient data [15], environmental data [16], and other data resources in the country [17, 18].

In this paper, we illustrate the potential of record linkage to better understand complex human traits to inform prevention, intervention, and policy by investigating environmental factors that potentially influence well-being. Most existing research on environmental effects for well-being to date follows a pick and choose approach e.g., [19–21], which could result in selective reporting or overestimation of effects. To overcome these limitations we propose a data-driven design, an *Environment-Wide Association Study* [22] (EnWAS). This approach is based on the principles of a genome-wide association study (GWAS), where each genetic marker in the genome is systematically tested for association with the phenotype of interest. Instead of genetic markers, EnWAS systematically associates environmental variables while reducing the chance of spurious findings by accounting for multiple testing. This data-driven approach is of particular interest given the lack of theoretical inclusion models and was recently successfully applied to examine behavioural patterns, psychosocial factors, mental and physical health conditions, access to and utilization of health care, and anthropometrics with physical, mental and social well-being [23]. From a broad range of psychosocial factors, 3 factors were associated with well-being: depressive symptoms, life satisfaction, and happiness. While this study provides us with valuable information of psychosocial associations with well-being, it did not explore physical environmental factors such as neighbourhood characteristics, in relation to well-being. Given that many governmental decisions and prevention and intervention policies are enroled at a neighbourhood level it is very important to get an indication of the effect of neighbourhood-level characteristics on person-level well- being.

In order to examine environmental variables associated with well-being, we applied EnWAS by linking well-being data from the population based Netherlands Twin Register (NTR) [17] to environmental data from the Geoscience and Health Cohort Consortium (GECCO) [16]. We examine 139 environmental variables that cover most aspects of people’s living environments e.g., land use in terms of build area or green space, and neighbourhood characteristics, such as safety and livability. In addition, given that it is widely accepted that people do not randomly choose where they live [24, 25], that differences in well-being are partly accounted for by genetic differences [26, 27], and to overcome possible genetic confounding, we use a genetically informative design. With this design we examine whether environmental exposure is driven by genetic predisposition for well-being. By combining exposome, phenome, and genome data, we aim to extend the limits of traditional approaches to get more comprehensive insight in how well-being can be placed in a broader context [28].

## Materials and methods

### Sample

This study used well-being data from the Adult sample of the NTR [17, 29]. For the current project, we made use of data collected in the 6th wave of data collection (2002/2003), and the 8th wave of data collection (2009/2010). These waves were chosen based on the fact that we collected satisfaction with life data at both these time-points. This resulted in a dataset of 9951 individuals for 2002/2003 and 11,975 individuals for 2009/2010. Sample characteristics can be found in Table 1. Depending on the missing-ness of environmental data per GECCO dataset, the number of individuals per analysis varies slightly across analyses.

**Table 1:** Sample characteristics.

Sample	n_individuals_	n_males_/n_females_	Mean age (range)	Mean SWL (SD)
2002/2003 Full Sample	9951	4158/5790*	39.4 (16–85)	26.6 (5.26)
2009/2010 Full Sample	11,975	4608/7363*	45.8 (16–97)	27.3 (5.18)
Polygenic score sample	7527	2602/4926	41.7 (16–90)	27.5 (5.18)
2002/2003 PC regression	5655	2603/3052	44.0 (16–85)	26.6 (5.22)
2009/2010 PC regression	4922	1702/3219**	48.5 (16–97)	27.33 (5.25)

*SWL* satisfaction with life.

*Age was unknown for 2 individuals.

**Age was unknown for 1 individual.

### Well-being data

To quantify well-being, we used the satisfaction with life (SWL) scale [30, 31]. The SWL scale consists of five items measuring satisfaction with life. Each item required a judgement of a given statement pertaining to SWL on a response scale ranging from 1 (strongly disagree) to 7 (strongly agree), summed to create individual sum scores ranging from 7 to 35.

### Environmental exposure data

**Fig. 1:**
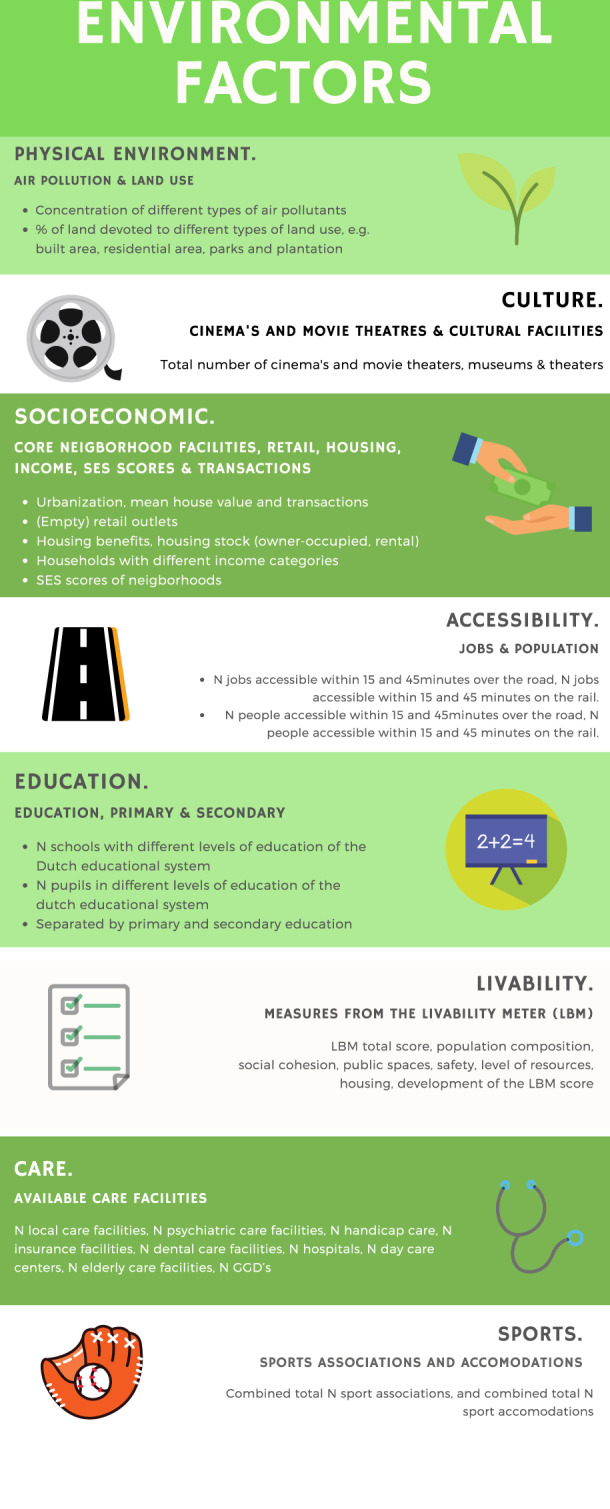
Overview of studied environmental domains. Environmental studies from the following domains were included: the physical environment, culture, socioeconomic, accessibility, education livability, care, and sports. SES socioeconomic status.

Environmental data were extracted from the Geoscience and Health Cohort Consortium (GECCO) [16] database. The GECCO database is a centralized collection of longitudinal geo-data on different geospatial levels in the Netherlands. As the GECCO data were collected in different time frames, we matched the GECCO data to the NTR data available in 2002/2003 and 2009/2010. In total, 1330 postal-code level variables are available within 34 predefined domains in the GECCO database (see Supplementary Tables S1 and S2). The data sources from which GECCO extracted the variables are frequently used government/census data resources, more information on which can be found in Supplementary Table S2. The environmental exposure data that are available in the GECCO database encompass a wide range of environmental domains, including social, physical and demographic variables. We selected variables representative of neighbourhood characteristics, regardless of which environmental domain they encompass. We pre-selected 168 variables based on two criteria: (1) availability at the same time-points as the NTR well-being assessment, and (2) we chose the most representative variables per domain to prevent inclusion of duplicate variables/ variables that were, without inspection of the data, expected not to vary across the Netherlands. Supplementary Table S3 provides an overview of these preregistered variables. Ultimately, quality control led to the inclusion of 133 variables grouped in 22 domains (see Supplementary Table S4). Importantly, some GECCO variables were assessed in both 2002/2003 and 2009/2010, and some exclusively at one of the time-points. More specifically, 80 variables were measured exclusively in 2002/2003, 23 variables were measured exclusively in 2009/2010, and 15 variables were measured on both occasions. Four-digit postal codes were used to link the environmental data to individual level well-being. Figure 1 and Table 2 describe the included domains, and Supplementary Table S5 provides descriptive statistics on these exposure variables. Since we also included educational attainment variables, we provide a schematic overview of the Dutch educational system in Supplementary Fig. S1.

**Table 2:** Overview of the included environmental domains.

Domains	Description
Accessibility	Data on accessibility include the total number of persons and jobs that are accessible within 15, 30, and 45 min over the road and on the rail.
Air pollution	Residential exposure to air pollutants was assessed as annual average concentrations of particulate matter with diameters of less than 2.5 µm, and between 2.5 µm and 10.0 µm, PM2.5 absorbance, and annual average concentrations of nitrogen oxides.
Cinema’s and movie theatres	Data on the number of cinema’s and movie theatres.
Facilities care	Data on the number of care-related facilities (e.g. hospitals, care homes).
Facilities culture	Data on the number of cinema’s, museums and theatres.
Facilities education	Data on the number of schools/educational locations and students stratified for level of education (see Fig. S7 for more information on the Dutch educational system).
Facilities retail outlets	Data on the number of retail outlets.
Facilities sport	Data on the number of a variety of sport facilities.
Housing benefits	Data on housing benefit receivers, accounting rent, accounting income, the height/sum of housing benefits.
Housing stock	Data on number/percentages of houses in the owner occupied sector, and (private and social) rental sector.
Income	Data on disposable income per person and household.
Core neighbourhood characteristics	Data on core neighbourhood characteristics, e.g. urbanization and mean house value.
Land use	Data on number of hectares that are related to specific land use (e.g. traffic, residential area).
Livability	Livability is measured using the “*leefbaarometer”* (LBM total score). Livability is defined as the extent to which the living environment is in line with the conditions and needs of residents. The LBM total score is based on six dimensions. These dimensions are: (1) population composition, (2) social cohesion, (3) public space, (4) safety, (5) level of resources, and (6) housing.
Museums, music theatres and pop podia	Data on number of museums, music theatres and pop podia.
Offices, retail and businesses	Data on number purchased and rented offices, retail and businesses. Data on the area of these buildings and related rental/sale costs are also available.
Primary education	Data on the number of primary schools and the number/percentages of pupils at these schools (Fig. S1).
Secondary education	Data on the number of schools with secondary education and the number/percentages of pupils at these schools (Fig. S1).
Socio-economic status scores	Data on socio-economic status scores based on education, income and position in the labour market.
Special education	Data on the number of schools with special education and the number/percentages of pupils at primary and secondary special schools.
Sport associations	Data on the number of hockey-clubs, baseball clubs, korfball clubs, tennis clubs, rugby clubs, and football clubs.
Transactions and average house prices	Data on the number of transactions, stratified for type of houses. In addition, the data-set includes data on the average house price.

### Genetic data

Genotyping was performed on different SNP micro-arrays that were cross-platform imputed using the Genome of the Netherlands (GoNL) reference set [32]. Quality control procedures are described in the Supplementary Methods. Principal component analysis (PCA) was performed to create genomic PCs reflecting ancestry and genotyping batch effects (for details see Abdellaoui et al. 2013). In total, genetic data and well-being scores were available for 7527 individuals (see Table 1).

### Analyses

This project was pre-registered at the open science framework (OSF) (https://osf.io/xehkc). Non-pre-registered follow-up analyses are indicated as such throughout the paper.

### Pre-registered

#### Regression analyses

We pre-registered multilevel models to account for potential within-postal code well-being similarity of participants. Supplementary Table S6 summarizes the number of participants per postal code. However, after accessing the data, the intra-class correlation (ICC) for well-being showed that the dependency of the observations within postal code is neglible (0.02 for 2002/2003 and 0.002 for 2009/2010). Therefore, we proceeded our analyses with generalized estimating equation (GEE) models, instead of multilevel models. GEE corrects for correlated observations, allowing us to include the full sample (instead of only genetically unrelated individuals). Regression analyses were performed for each environmental predictor, with sex, age, and age-squared as covariates. Familial relatedness was accounted for using an exchangeable conditional covariance matrix based on sandwich-corrected standard errors [33], as implemented in the GEE package in R. Statistical significance was assessed using a Bonferroni-corrected significance threshold of 3.6 × 10^−4^ (0.05/139). Power to detect associations with different potential effect sizes can be found in the pre-registration.

#### Polygenic risk score analysis

To assess the role of genetic factors in the associations obtained in the GEE analyses, we performed polygenic score (PGS) prediction analyses. A PGS reflects an individual’s genetic liability for a trait of interest, calculated from the effect sizes from GWA summary statistics. The PGSs were computed for the well-being spectrum in NTR participants using the GWA summary statistics (recomputed excluding NTR) from Baselmans et al. [34]. The summary statistics were recomputed using LDpred [35]. These recomputed summary statistics were turned into PGSs using allelic scoring function in PLINK [36]. This function aggregates the number of effect alleles weighted by their effect estimates in each individual to create scores reflecting an individual’s genetic liability for a trait. GEE was used to test the association of the well-being spectrum PGSs (independent variable) with significant environmental correlates (dependent variables) from the EnWAS. Age, age-squared, sex, and the first ten genomic PCs were included as covariates.

In addition, we used the well-being spectrum PGSs to split the sample into septiles to evaluate the potential of stratifying individuals based on a PGS for well-being. The first septile contains participants with the lowest genetic susceptibility for well-being, and the seventh septile contains those with the highest. We calculated the mean well-being and environmental value per septile and compared whether these means differed significantly by examining overlap in confidence intervals.

### Non pre-registered

#### Multicollinearity follow-up

In the univariate analyses the covariates were considered one at a time, thus ignoring the possible correlation between these variables. To illustrate the overlap between the different variables that significantly predict well-being, we visualized the correlations in chord diagrams using the circlize package in R [37]. We plotted the associations separately for the variables from 2002/2003 and 2009/2010, and made separate plots for: (a) correlations stronger than 0.8, and (c) correlations stronger than 0.4.

Next, to accommodate the relative strong correlations between the environmental factors (see Supplementary Table S7), we ran a principal component analysis (PCA) of the standardized environmental exposures using the prcomp function from the stats package in R. We aimed to extract independent principal components (PCs) that explained at least 90% of the environmental data. Next, these uncorrelated PCs were used as independent predictors to predict well-being in an unrelated sample (after the effects of age, age [2], and sex were regressed out). Based on this analysis, we examined how much variance in well-being can be explained by the combined environmental factors.

#### Socioeconomic status correction

In the exploratory, data-driven approach of our initial pre-registered analyses, we did not correct for socioeconomic status (SES). However, outcomes of the GEE and the principal component analyses suggested a potential role of SES in the associations. Therefore, as none-preregistered follow-up, we repeated the GEE analyses while correcting for SES using two strategies: (1) including the individual’s educational attainment to approximate individual SES, and (2) including the GECCO variable “status score of the neighbourhood” as a measurement of neighbourhood SES (see Supplementary Methods for more information).

## Results

### Regression analyses

**Fig. 2:**
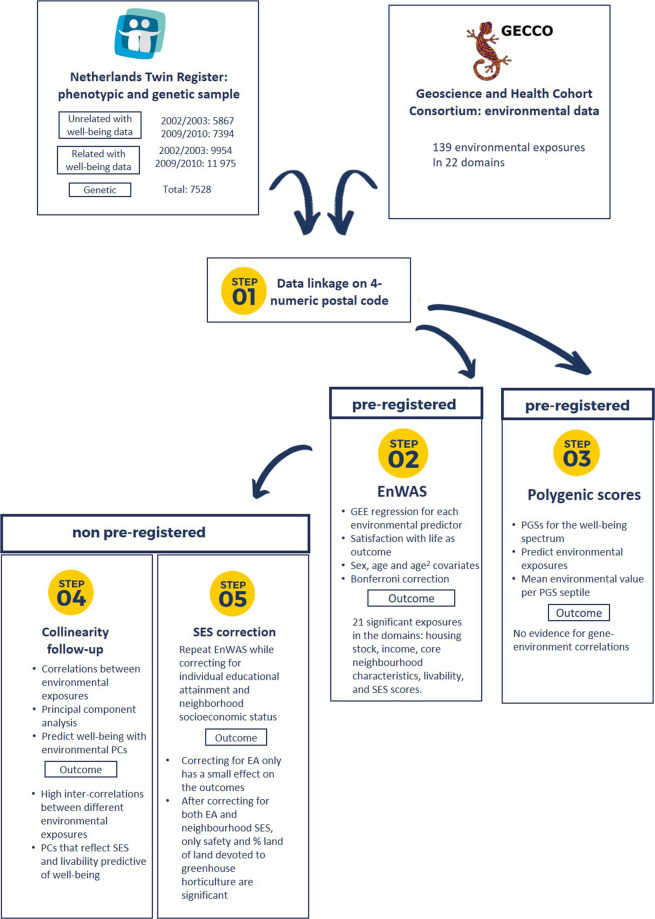
Overview of performed analyses and results. EnWAS environment-wide associations study, GEE generalized estimating equation, PGS polygenic scores, PC principal component, SES socioeconomic status, EA educational attainment.

**Fig. 3:**
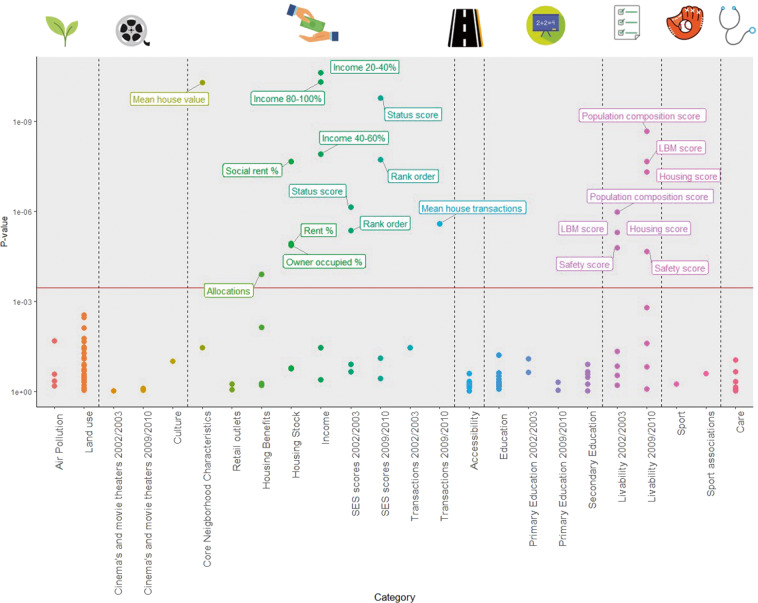
Manhattan plot showing *P* values for associations between environmental variables within different categories and well-being. Significance is indicated with the red line representing the *p*-value threshold corrected for multiple testing. Individual variable descriptions can be found in Supplementary Tables 1–3.

In the GEE analyses, 21 of the 139 environmental variables passed the Bonferroni-corrected threshold and thus were found to be associated with well-being (Figs. 2–3, and Table 3). These variables were included in the domains: housing stock, income, core neighbourhood characteristics, livability, and SES scores. An overview of all associations can be found in Supplementary Table S8.

**Table 3:** Significant associations with well-being from the generalized estimation equation (GEE) analyses.

Domain	Variable	*β* (*SE*) GEE	*P* value GEE	*R*^*2*^ GEE
Housing Benefits	Housing benefits (allocations)	−0.045 (0.01)	1.2 × 10^−4^	0.002
Housing Stock	Social rental sector %	−0.066 (0.01)	2.14 × 10^−8^	0.004
Housing Stock	Rental sector %	−0.051 (0.01)	1.15 × 10^−5^	0.003
Housing Stock	Owner occupied %	0.051 (0.01)	1.31 × 10^−5^	0.003
Income	Income 80–100%	0.069 (0.01)	4.89 × 10^−11^	0.005
Income	Income 20–40%	−0.076 (0.01)	2.37 × 10^−11^	0.006
Income	Income 40–60%	−0.059 (0.01)	1.21 × 10^−8^	0.003
Core neighbourhood characteristics	Mean house value	0.064 (0.01)	5.21 × 10^−11^	0.004
Livability 2002/2003	Population composition	0.057 (0.01)	1.05 × 10^−6^	0.003
Livability 2002/2003	Livability (LBM) score	0.053 (0.01)	5.00 × 10^−6^	0.003
Livability 2002/2003	Housing score	0.052 (0.01)	5.02 × 10^−6^	0.003
Livability 2002/2003	Safety score	0.049 (0.01)	1.60 × 10^−5^	0.002
Livability 2009/2010	Livability (LBM) score	0.059 (0.01)	2.19 × 10^−8^	0.003
Livability 2009/2010	Housing score	0.057 (0.01)	4.91 × 10^−8^	0.003
Livability 2009/2010	Population composition	0.064 (0.01)	2.16 × 10^−9^	0.004
Livability 2009/2010	Safety score	0.044 (0.01)	2.12 × 10^−5^	0.002
SES scores 2002/2003	Status score	0.057 (0.01)	7.01 × 10^−7^	0.003
SES scores 2002/2003	Rank order	−0.052 (0.01)	4.30 × 10^−6^	0.003
SES scores 2009/2010	Status score	0.067 (0.01)	1.68 × 10^−10^	0.004
SES scores 2009/2010	Rank order	−0.057 (0.01)	1.90 × 10^−8^	0.003
Transactions 2009/2010	Mean house transactions	0.051 (0.01)	2.52 × 10^−6^	0.003

*SES* socioeconomic status, *β* beta, *SE* standard error, *GEE* generalized estimation equation, *R*^*2*^ R-squared.

### Polygenic risk score analysis

The well-being spectrum polygenic score predicted well-being in our sample (*R*^2^ = 0.007, *P* = 5.11 × 10^−12^), but it did not predict any of the environmental correlates (Table 4). In addition, no mean difference between polygenic septiles was observed for any of the variables (see Supplementary Table S9).

**Table 4:** Associations well-being polygenic score with environmental exposures.

Domain	Variable	PGS *β* (*SE*)	PGS *P* value
Housing Benefits	Housing benefits (allocations)	−0.020 (0.02)	0.246
Housing Stock	Social rental sector %	−0.017 (0.02)	0.310
Housing Stock	Rental sector %	−0.002 (0.02)	0.929
Housing Stock	Owner occupied %	0.002 (0.02)	0.929
Income	% people income 5th percentile	0.007 (0.02)	0.672
Income	% people income 2th percentile	0.002 (0.02)	0.906
Income	% people income 3th percentile	−0.006 (0.02)	0.704
Core neighbourhood characteristics	Mean house value	0.009 (0.02)	0.560
Livability 2002/2003	Population composition	0.030 (0.02)	0.066
Livability 2002/2003	Livability (LBM) score	0.030 (0.02)	0.075
Livability 2002/2003	Housing score	0.012 (0.02)	0.467
Livability 2002/2003	Safety score	0.011 (0.02)	0.517
Livability 2009/2010	Livability (LBM) score	0.015 (0.02)	0.313
Livability 2009/2010	Housing score	0.019 (0.02)	0.211
Livability 2009/2010	Population composition	0.017 (0.02)	0.278
Livability 2009/2010	Safety score	0.008 (0.02)	0.614
SES scores 2002/2003	Status score livability	0.017 (0.02)	0.289
SES scores 2002/2003	Rank order livability	−0.014 (0.02)	0.397
SES scores 2009/2010	Status score livability	−0.011 (0.02)	0.477
SES scores 2009/2010	Rank order livability	0.006 (0.02)	0.691
Transactions 2009/2010	Mean house transactions	0.016 (0.02)	0.334

*SES* socioeconomic status, *PGS* Polygenic Score, *β* beta, *SE* standard error.

### Multicollinearity follow-up

Strong correlations (ranging between −1 and 0.87) were observed between the significant variables from the GEE analyses (Supplementary Table S7). For both time-points, we plotted the variables that were correlated 0.8 or stronger (Supplementary Figs. S2A and S3A), and .4 or stronger (Supplementary Figs. S2B and S3B) using chord plots. These plots display all associations (above our defined thresholds) between the included variables. The variables are presented in a circle, and whenever a line connects two variables, it indicates they are associated. For both time-points, when we defined the threshold as correlations >0.4, we see that all variables are connected to all other variables, creating a densely connected plot. However, when we increased the threshold to 0.8, the plots become more organized with only few connections remaining. For the 2002/2003 data, this resulted in a plot with three clusters: (1) a housing cluster with housing score, housing stock owner-occupied, housing stock: rental, and housing stock: social rent, (2) a livability cluster of livability scores, population composition scores, and safety scores, and (3) another livability cluster with status scores and rank order of the neighbourhoods. For the 2009/2010 data, we see two clusters: (1) an SES cluster including two income variables, mean house value, and the status score and rank order of the neighbourhood, and (2) a livability cluster including LBM scores, population composition, and safety.

The PCA extracted 95 and 38 independent PCs for 2002/2003 and 2009/2010, respectively. The first 43 PCs cumulatively explained 90.5% of the 95 environmental variables in the 2002/2003 data, and the first 16 PCs explained 90.7% of the 38 environmental variables in the 2009/2010 data (see Supplementary Table S10). Combined in one linear regression model, these 43 PCs explained 1.45% of the variance in well-being in the 2002/2003 data. After correcting for the number of PCs included, this decreased to 0.69% (adjusted *R*^2^). One PC (PC3: *β* = −0.029, *SE* = 0.006, *P* = 2.73 × 10^−7^) significantly predicted well-being after correcting for multiple testing. For the 2009/2010 data, the 16 PCs explained 1.11% of the variance in well-being, which decreased to 0.79% after correcting for the number of PCs (adjusted R^2^). Two PCs significantly predicted well-being (PC1: *β* = 0.0185, *SE* = 0.005, *P* = 0.0001, PC2: *β* = −0.0240, *SE* = 0.006, *P* = 3.4 × 10^−5^). Supplementary Table S11 lists the environmental variables with loadings higher than 0.1 with the significant PCs. For the 2002/2003 data, the PC that significantly negatively predicted well-being was represented by four variables reflecting low-income neighbourhoods. For the 2009/2010 data, one of the PCs (PC1) was indicative of high income and livability, while the other PC (PC2) was indicative of low income and lower livability.

### Analyses with socioeconomic status

Correcting for individual EA had a small effect on the observed associations. After also including the SES of the neighbourhood, only neighbourhood safety and the percentage of land devoted to greenhouse horticulture remained significant (see Supplementary Table S8).

A summary of all analyses and their results can be found in Fig. 2.

## Discussion

The present study linked two large data-resources in the Netherlands in order to examine potential associations between well-being and a range of environmental factors. Using this environment-wide association approach, we identified 21 environmental factors that were associated with well-being. These factors cluster in the following domains: housing stock, income, core neighbourhood characteristics, livability, and SES. A common theme that emerged is that the identified correlates can be classified as socioeconomic indicators.

An examination of the correlations between these variables reveals that they are not independent. When correcting for individual and neighbourhood SES, only safety and % of land devoted to greenhouse horticulture were significantly associated with well-being, with safer neighbourhoods and neighbourhoods with more greenhouse horticulture showing higher average levels of well-being. A closer examination of the distribution of these two environmental variables in the Netherlands (Supplementary Figs. S4–6) revealed that greenhouse horticulture did not show a lot of variation across the country, especially compared to the other associated variables (SES and safety). Therefore, this association should be interpreted with caution. Safety, on the other hand varies widely across the different postal codes. Earlier studies also found associations between psychological health and neighbourhood safety [38–40]. It is furthermore in line with previous research where well-being was linked to neighbourhood-level SES indicators [41, 42]. Moreover, similar results have been found for depression using GECCO data [43]. Importantly, what should be kept in mind when examining the results of this study is that we are examining associations, and not causal effects. For the identified associations, this means two things should be considered. First, there might be some third, mediating factor that explains the associations. Most of the factors assessed in the first round of EnWAS disappeared when we corrected for SES, already suggesting that SES was driving these associations. Secondly, even if there are potential causal associations, we cannot make any statement regarding the direction of the effect.

No effects of genetic differences were observed, indicated by the absence of significant genetic prediction. This indicates that either the genetic predisposition for well-being does not cause individuals to pick certain environments or that we suffer from a lack of power. Indeed, a post-hoc power analysis (Supplementary Fig. 7) indicates that with our current sample size and alpha, we could have detected associations between the well-being polygenic scores and environmental exposures with effect sizes greater than *R*^2^ = 0.002. Thus, associations between the current PRS and the environmental exposures assessed here are likely extremely small. The well-being spectrum polygenic score explains less than a percentage of the variance in well-being itself, and there was no difference in mean well-being between different genetic susceptibility groups. This raises the question of whether a stronger PGS would lead to different results than presented here. Therefore, while any statement on this genetic component is speculative at this moment, we cannot write off the potential role that genetics play in these associations, and encourage future investigations in this area.

From the existing literature, we already knew that the effect of individual genetic variants on well-being is small [44]: 12-18% of the variance in well-being is explained by ~600k genome-wide measured SNPs for complex traits, with GWA study SNP-based heritability estimates around ~5% [27]. Here, we report small environmental effects on well-being. The significant environmental predictors from the EnWAS individually explain only 0.2% to 0.5% of the variance in well-being. In addition, the PCA showed that the combined effect of the EnWAS variables explains only around ~1% of the variance in well-being. Important to keep in mind while interpreting these effect sizes is the fact that we examined environmental exposures at the postal code level. It is likely that the well-being exposome varies over different geographical levels (e.g., cities, municipalities) [45], measures of well-being, and is differently associated with subjective indicators of the environment [46, 47]. Take as an example SES: studies examining the effect of individual-level SES on well-being find estimates as large as 6% explained variance [48, 49], which is much larger than our current finding for neighbourhood SES indicators.

Moreover, we did not, despite our large sample, find any evidence for many previously suggested indicators, such as the presence of green space [50] or air pollution [51]. Different reasons might explain this discrepancy: e.g., the level and country of examination (postal code level in the Netherlands), the use of objective indicators of the environment (instead of subjective experiences), and the measure of well-being we used. Therefore, our findings should be interpreted in the context of this study. Important, though, is that our study investigates the association between wellbeing and postal code linked variables, e.g., the amount of greenspace in the postal code area. That is a different approach than studying wellbeing in relation to frequency of visiting or enjoying greenspace. In order to develop a full picture of the well-being exposome, it is necessary to take these different aspects into account. Mapping the well-being exposome will also require investigations on different time-points or, optimally, longitudinal investigations tracking the dynamic interplay and direction of causality between environmental factors, biological factors and well-being [52]. For consistency, we decided to assess each variable on the same geospatial scale (PC-4 level). However, this level is likely not the most relevant level for each assessed exposure variable. The methodology used in this project can easily be applied to different levels of analysis (e.g. individual level objective data, individual level subjective data, street level). In this way, we can compare EnWAS results on different levels, offering a replicable means of mapping the well-being exposome. What should additionally be kept in mind is that many studies focus their efforts on one or a few exposures at a time, limiting the potential to study such an exposure in a broader context. This study demonstrates the importance of large, data-driven explorations to get a more adequate image of these intertwined environmental associations.

In the genetics field, small effects are common and combined in polygenic scores that are used for more in-depth analyses. An interesting approach would be to combine environmental effects in “*poly-environmental*” scores. In this way, small environmental effects can be combined and used to predict well-being. An obstacle that needs to be overcome in order to construct these scores is that we need a better understanding of the correlational structure between different environmental factors. In case of polygenic scores, we can correct for correlations between genetic variants based on our knowledge of recombination patterns and linkage disequilibrium [35]. For poly-environmental scores, however, the association between different environmental factors is much more complex and dynamic. By combining small effects in poly-environmental scores, complemented by polygenic scores, it might in the future be possible to develop personalized prevention and intervention strategies for well-being. However, in addition to acquiring better knowledge of the correlational structure of the environment, this will also require more insight into the potential direction of causality of current findings. Another interesting direction for future research that aims to combine genetic and environmental effects is to compare the well-being of monozygotic twins that are exposed to different living environments. Since monozygotic twins are 100% genetically identical, a difference in well-being between the twins can only be caused by unique environmental experiences. Therefore, by associating monozygotic intra-pair difference scores for well-being with intra-pair difference scores for environmental exposure, it becomes possible to examine the extent to which an association between well-being and an environmental exposure exists independent from genetic and shared environmental factors. In our sample, there was a relatively low number of *complete* monozygotic twin pairs for which we could compute difference scores for both well-being and the environmental exposures (*N*_pairs_ 2002/2003 = 389, *N*_pairs_ 2009/2010 = 270). As a result, no evidence was found for an association between well-being intra-pair difference scores and any of the environmental exposure intra-pair difference scores (see Supplementary Table S12). Therefore, we encourage other cohorts with larger samples to perform these analyses in order to get a grasp of the potential genetic effects.

To conclude, in this study we combined the strengths of record linkage to understand individual differences in well-being. Taken together, our analyses suggest that, at the postal-code level, the most important predictors of well-being are socioeconomic factors and safety. Moreover, we find that environmental effects are typically small and context dependent, emphasizing the need for large scale linkage efforts and data-driven designs.

## Supplementary information

Supplementary Tables

Supplementary Methods

Supplementary Material

Supplementary Figures

## Data Availability

The Netherlands Twin Register cohort data may be accessed through the Netherlands Twin Register (ntr.fgb@vu.nl) upon approval of the data access committee. The environmental exposure data may be accessed through the Geoscience and Health Cohort Consortium (gecco@vumc.nl) upon approval of the data access committee.
