# Supplementary material for: Expanding the environmental scope: an environment-wide association study for mental well-being
Source: J Expo Sci Environ Epidemiol. 2021 Jun 14;32(2):195–204. doi: 10.1038/s41370-021-00346-0 (PMC8920882; doi:10.1038/s41370-021-00346-0)
Supplement: Supplementary file 2 — Supplementary Methods [file 41370_2021_346_MOESM2_ESM.docx]

**Supplementary Methods**

*Genetic quality control (QC) procedures*

QC procedures included the removal of SNPs with allele frequency differences with the reference set of >10%, minor allele frequency (MAF) <.005, deviation from Hardy-Weinberg Equilibrium (HWE) with p < 10−12, and genotyping call rate <.095. Additionally, we removed samples with genotyping call rate <.09, inbreeding coefficient < -.075 / >.075., Affymetrix contrast QC metrix <.40, Mendelian error rate >5 SD from the mean, and identity-by-state status that did not match know relationship status/genotypic assessment were excluded. After phasing and imputation using the MaCH-Admix software41 we performed post-imputation QC as described in Baselmans et al (Baselmans et al., 2019).

*Educational attainment and socioeconomic status correction*

We corrected in two ways: first, to approximate individual SES, we included a variable representing the individual’s educational attainment (EA). EA was scored in seven categories: 1) primary school only, 2) lower vocational schooling, 3) lower secondary schooling, 4) intermediate vocational schooling, 5) intermediate/higher secondary schooling, 6) higher vocational schooling and 7) university. This EA variable was included as a covariate in all 139 associations. In addition to EA, we included the GECCO variable “status score of the neighbourhood” as a measurement of neighbourhood SES. These status scores were created based on three variables: 1) the percentage of individuals with a low income, 2) the percentage of individuals with low EA, and 3) the unemployment rate within the postal code. We did not include the SES variable in the association analyses with variables in the domains income and SES scores, as these are used to calculate the SES variable. Therefore, we corrected for neighbourhood SES in 132 associations.

**References**

Baselmans, B. M. L., van de Weijer, M. P., Abdellaoui, A., Vink, J. M., Hottenga, J. J., Willemsen, G., Nivard, M. G., de Geus, E. J. C., Boomsma, D. I., & Bartels, M. (2019). A Genetic Investigation of the Well-Being Spectrum. *Behavior Genetics*, *49*(3), 286–297. https://doi.org/10.1007/s10519-019-09951-0
