# Supplementary material for: Expanding the environmental scope: an environment-wide association study for mental well-being
Source: J Expo Sci Environ Epidemiol. 2021 Jun 14;32(2):195–204. doi: 10.1038/s41370-021-00346-0 (PMC8920882; doi:10.1038/s41370-021-00346-0)
Supplement: Supplementary file 3 — Supplementary Material [file 41370_2021_346_MOESM3_ESM.docx]

Supplementary Material:

Figure S1: Overview of the educational system in the Netherlands

Figure S2*.* Correlations between variables that predict well-being. A) Correlations stronger than .8; B) Correlations stronger than .4 in 2002/2003.

Figure S3*.* Correlations between variables that predict well-being A) correlations stronger than .8; B) correlations stronger than .4 in 2009/2010.

Figure S4: Distribution of greenhouse horticulture in the Netherlands (in z-scores).

Figure S5: Distribution of safety scores in the Netherlands (in z-scores).

Figure S6: Distribution of socioeconomic status scores in the Netherlands (in z-scores).

Figure S7: Power analysis for the polygenic score prediction analyses with our given sample size of N=7527, and an alpha of .002.

Supplementary Excel File: Supplementary Tables

Supplementary methods file: Supplementary Methods
