# Supplementary material for: Expanding the environmental scope: an environment-wide association study for mental well-being
Source: J Expo Sci Environ Epidemiol. 2021 Jun 14;32(2):195–204. doi: 10.1038/s41370-021-00346-0 (PMC8920882; doi:10.1038/s41370-021-00346-0)
Supplement: Supplementary file 4 — Supplementary Figures [file 41370_2021_346_MOESM4_ESM.docx]

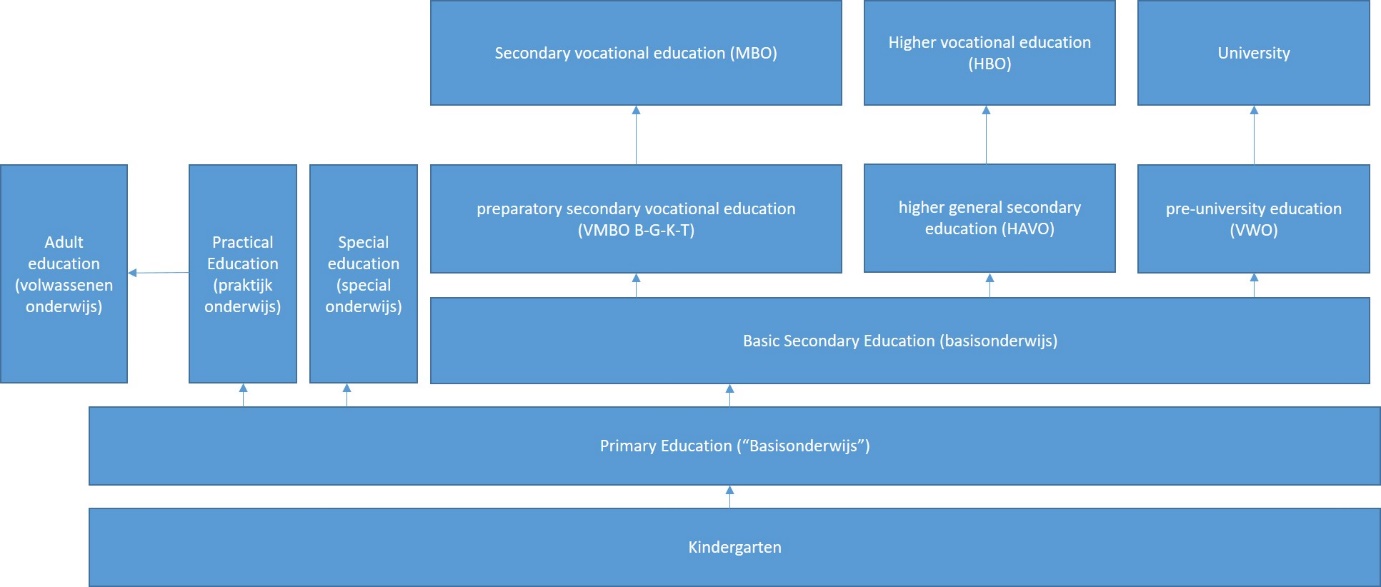


Supplementary Figure S1.


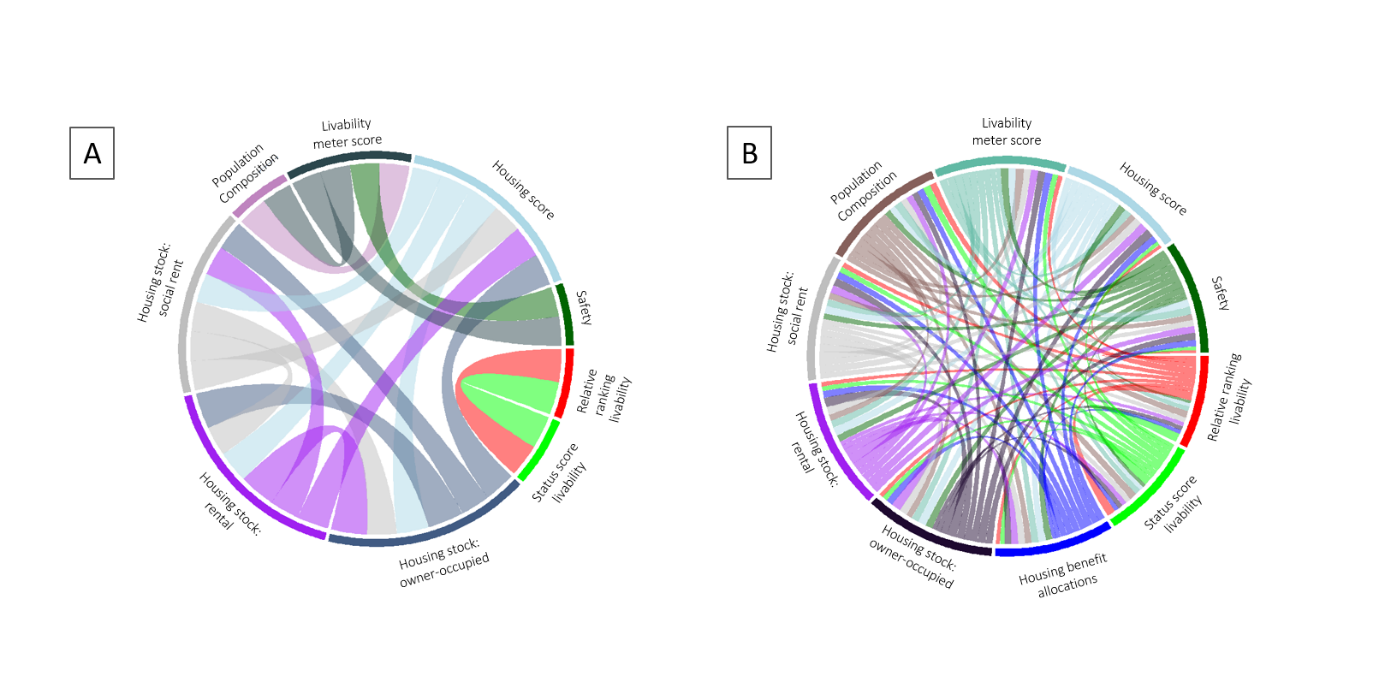


Supplementary Figure S2.


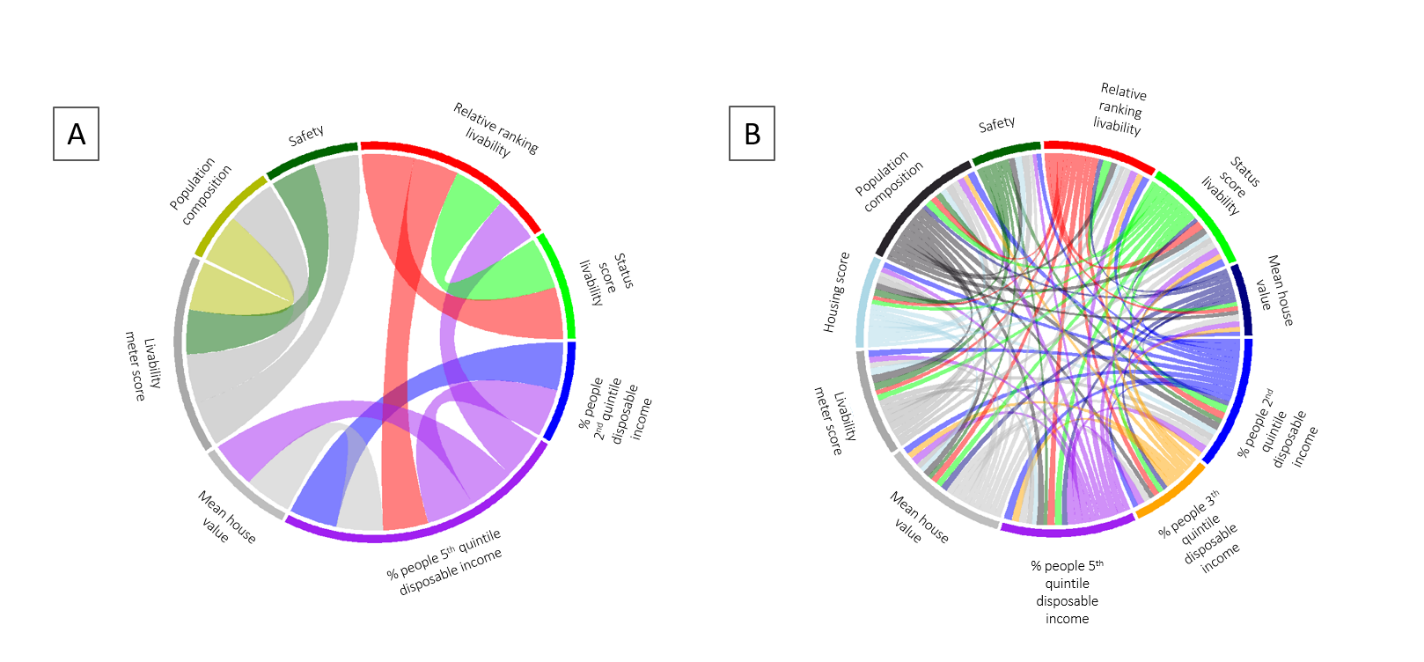


Supplementary Figure S3.

Supplementary Figure S4:

Supplementary Figure S5.

Supplementary Figure S6.


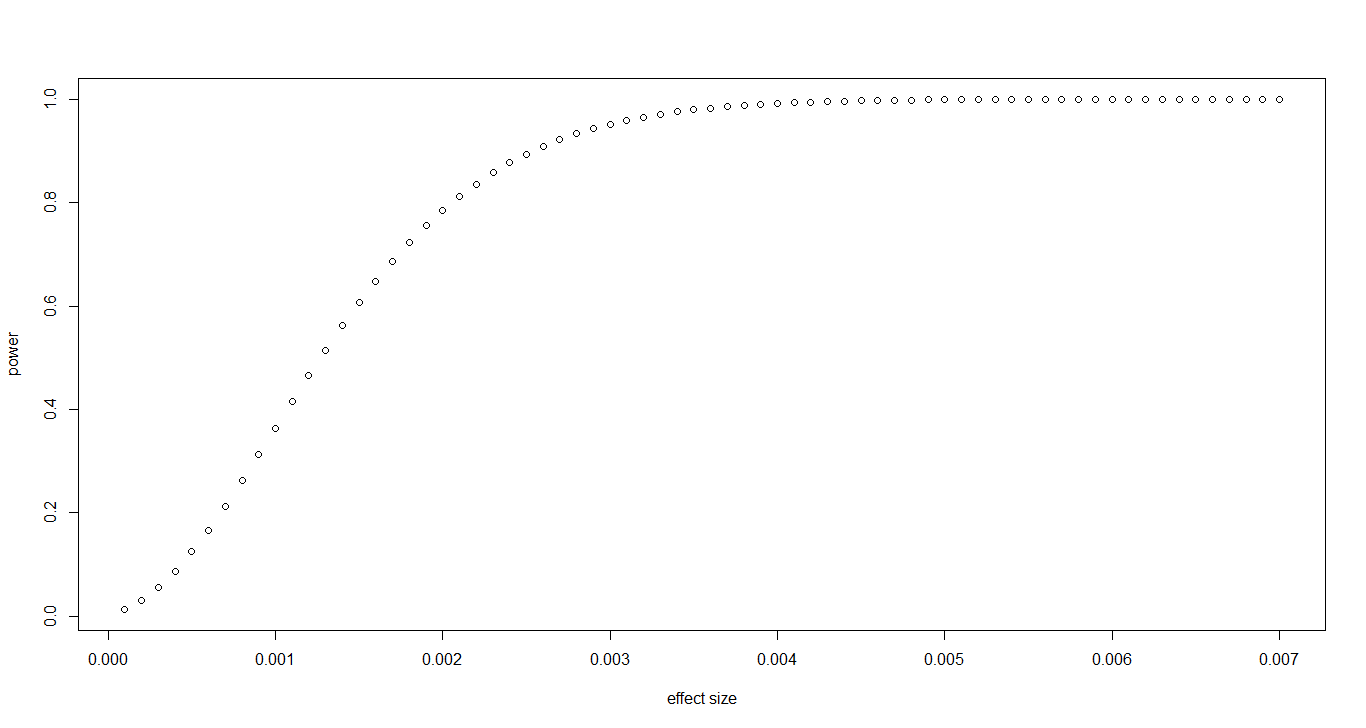


Supplementary Figure S7.
